# Deciphering the Arterial and Venous Blood Bacterial DNA Profile: Pioneering Insights into Coronary Heart Disease Etiology and Progression

**DOI:** 10.3390/microorganisms14020359

**Published:** 2026-02-03

**Authors:** Mengru Liu, Lin Zhao, Tianli Li, Xuelin Li, Hong Jiang, Peng Yang

**Affiliations:** Department of Integrative Medicine Cardiology, China-Japan Friendship Hospital, Beijing 100029, China

**Keywords:** metagenomics, microbial diversity, coronary heart disease, 16S rRNA sequencing, microbiota

## Abstract

Background: Coronary heart disease (CHD) is the leading cause of death and disability worldwide. The human microbiota, particularly gut bacteria, plays a role in the development of CHD. However, determining the contribution of gut bacteria translocation to systemic circulation in the progression of atherosclerosis remains challenging. Methods and Results: In this exploratory study, we conducted 16S rRNA–based metagenomic analysis to characterize systemic bacterial profiles in a cohort of 27 patients with CHD (9 with severe coronary artery stenosis and 18 with mild to moderate stenosis). We compared microbial diversity between arterial and venous blood and across different blood fractions. For the first time, we observed higher microbial diversity in plasma than in serum. We also identified differences in microbial richness among arterial whole blood, venous whole blood, arterial plasma, venous plasma, arterial serum, and venous serum, with 15, 22, 43, 10, 4, and 3 genera showing significant differences, respectively. Many of the detected blood taxa belonged to genera typically found in intestinal, oral, or skin microbiota, although their precise source cannot be determined from this study. Conclusions: Our study provides preliminary evidence of distinct bacterial profiles between arterial and venous blood fractions in patients with CHD, as determined by 16S rRNA sequencing. These findings should be interpreted with caution given the small sample size and the absence of a healthy control group, and they warrant confirmation in larger, controlled studies.

## 1. Introduction

Coronary heart disease (CHD) is the leading cause of death and disability worldwide [1]. Its risk factors include obesity, diabetes, and hyperlipidemia. While drug therapy and mechanical reperfusion have diminished CHD’s acute mortality rates, subsequent cardiovascular events continue to signify increased mortality post-treatment [2]. Notably, inflammation has been implicated in post-infarction cardiovascular remodeling and subsequent heart failure [2,3]. The inflammatory reaction triggered by CHD not only initiates cardiac repair but leads to remodeling and cardiac dysfunction [4,5]. Despite limited focus on elucidating the pathological mechanisms linking post-treatment inflammation to cardiovascular events, optimal therapeutic strategies are still scarce. The human body harbors tens of trillions of microorganisms, including bacteria, viruses, archaea and fungi [6]. The inflammatory response caused by the microbiome can affect the progression of CHD. Additionally, they are integral to nutrient metabolism, processing proteins, carbohydrates, dietary fibers, essential amino acids, and choline. Metabolites produced by the microbiome, such as short-chain fatty acids (SCFAs), bile acids (BAs), and trimethylamine N-oxide (TMAO), contribute to host immunity and metabolic regulation.

Recent studies indicates that gut microbiota play critical role in the development of chronic diseases, such as inflammatory bowel disease, liver cirrhosis, arthritis, and type 2 diabetes [7,8,9,10]. Atherosclerosis is also demonstrated to be promoted by intestinal metagenome and microbial metabolism [11]. Recent investigations have also linked disordered gut microbial communities to increased susceptibility to myocardial infarction (MI) in rat models [12]. Moreover, comparative studies reveal that symptomatic atherosclerotic plaques harbor a higher prevalence of pathogenic microorganisms, such as *Helicobacter pylori*, *Neisseria*, and *sulfur-oxidizing bacteria*, than those in asymptomatic patients [13], suggesting the significant role of bacterial translocation in atherosclerosis development.

As molecular biology advances, the association between blood microorganisms and various diseases has emerged as a significant research focus. The blood circulation is a closed system, and the blood of a healthy person is generally regarded as sterile [14,15]. However, studies have confirmed the presence of microorganisms in the blood of healthy individuals [16]. However, the concept of a “blood microbiome” remains highly controversial. Recent reviews and population-based studies have questioned the existence of a consistent endogenous blood microbiome and emphasized that many signals in low-biomass samples may reflect contamination or transient translocation rather than a stable microbial community [17,18,19,20]. The seminal work by Domingue et al. in 1977 marked the first documented report of bacteria in the blood of healthy individuals, with a prevalence of approximately 7% [21]. A study by Nikkari et al. in 2002 detected the bacterial 16S rRNA detected in blood samples of healthy individuals by PCR [22]. A study by Pretorius et al. in 2014 demonstrated the visualization of these samples under an electron microscope [23]. Recent studies obtained the specific blood microbial DNA profiles by testing patients with liver cirrhosis and cardiovascular disease in obese patients [24,25]. Patients suffering from MI exhibited 1.3 times higher plasma bacterial 16S rRNA levels than those individuals without coronary heart disease, along with a decrease in microbiota diversity [26]. Following experimental MI, the intestinal permeability was enhanced [27]. The gut barrier exerts to limit intestinal bacteria and toxic mediators escaping from the gut, thus avoiding a systemic inflammatory response [28]. Gut barrier breakdown leads to intestinal permeability increase, bacteria and endotoxin translocation into the systemic circulation, and immune-inflammatory system activation [29]. Disruption of intestinal barrier integrity and subsequent microbial translocation are now recognized as central mechanisms not only in inflammatory bowel disease but also in extraintestinal inflammatory disorders, where gut-derived microbial products contribute to chronic systemic inflammation and disease progression [30]. However, the samples tested in the study was obtained from patients’ venous blood. The characteristics of arterial blood microbes and their metabolites in patients with atherosclerosis, as well as the differences in microbial characteristics of venous blood, have not yet been studied. Blood flows from the pulmonary circulation to the systemic circulation. There are notable disparities in pressure, oxygen content, and metabolites between arterial and venous blood. Analysis of arterial and venous blood from 20 patients undergoing coronary artery bypass surgery with severe heart disease found that the species and levels of most miRNAs were similar, while the expression of a small number of miRNAs was different [31]. Consequently, in atherosclerosis patients, arterial blood composition varies to some degree. However, the distinction in microbial characteristics between arterial and venous blood remains unverified, necessitating additional investigation into blood microorganisms.

## 2. Methods

### 2.1. Study Cohort and Patient Characteristics

The patients admitted to China-Japan Hospital Heart Center from September to October 2020 in our previous work were enrolled. Based on coronary angiography results, patients were divided into two groups: a severe coronary artery stenosis group (lumen area reduction of 76–100%) and a mild-to-moderate stenosis group (lumen area reduction of 50–75%). The diagnosis and treatment of CHD followed the recently published guidelines [32]. Patients who had infectious or inflammatory disorders in the past six months or had received antibiotics, probiotics, or hormone-replacement therapy within the preceding eight weeks were excluded. All patients with severe stenosis underwent aspirin therapy prior to primary percutaneous coronary intervention (PCI), which was performed using conventional techniques with unrestricted coronary stent deployment. The infarct-related artery was the only target for the procedure. Finally, the study cohort comprised 9 patients with severe coronary artery stenosis and 18 with mild to moderate stenosis. The admission blood samples were collected after initial diagnosis and prior to the first stent intervention and antiplatelet medication. The study was performed in accordance with the Helsinki declaration and was approved by the hospital Research Ethics Committee. Informed consent was obtained from study participants. All blood samples were collected by trained medical staff using sterile, single-use needles and tubes under standard aseptic conditions.

### 2.2. Whole Blood and Blood Fractions Sample Preparation

Two EDTA blood tubes and two inert separating gelatinizing tubes (4 mL each per donor) were collected, verified for safety (tested negative for hepatitis B and C, HIV, AIDS and syphilis), typed, and transported at 4 °C. For each donor, arterial blood was obtained from the radial artery and venous blood from the antecubital vein using separate tubes. Inert separating gel tubes were then used for serum preparation from both arterial and venous blood, followed by centrifugation at 2500× *g* for 15 min at 4 °C. Arterial and venous blood samples from each donor were collected into EDTA tubes for plasma preparation. Following centrifugation at 2500× g for 15 min at 4 °C, the plasma was extracted. For whole blood, subfractions were meticulously prepared in a Class II biological safety cabinet.

### 2.3. DNA Extraction from Whole Blood, Serum and Plasma Samples

The protocols of DNA extraction were carefully designed to minimize contamination risk between samples or from the experimenters. DNA was extracted from 200 µL whole blood fractions using the NucleoSpin Blood Kit (Macherey-Nagel, Düren, Germany). The process involved three 30 s mechanical lysis cycles at 30 Hz in a bead beater (TissueLyser, Qiagen, Hilden, Germany) with 0.1 mm glass beads (Mo Bio Laboratories, Inc., Carlsbad, CA, USA), enhancing bacterial DNA yield. The quality and quantity of extracted DNA were monitored by gel electrophoresis (1% agarose in TBE 0.53) and ultraviolet spectrophotometer (Nano-Drop 2000, Thermo Scientific, Wilmington, DE, USA). DNA from plasma (240 uL) and serum (240 uL) were extracted using a DNA isolation kit (NucleoSpin Plasma XS, Macherey-Nagel, Düren, Germany) and stored all DNA extracts at −20 °C until further processing. Because this was an exploratory low-biomass study, no reagent-only negative extraction controls or sequencing blanks were included in the workflow.

### 2.4. 16S rRNA Gene Quantification by Real-Time qPCR

The concentration of 16S rRNA gene copies were normalized to 1 mL of whole blood in each sample. Subsequently, the V3 and V4 region of the bacterial 16S rDNA gene was amplified by PCR using primers: EUBF 50-TCCTAC GGGAGGCAGCAGT-30 and EUBR 50-GGACTACCAGGGTATCTAATCCTGTT-30 [33]. The primers exhibit high sensitivity (targeting 95% of the bacterial sequences found in the Ribosomal Database Project) and 100% specificity (no eukaryotic, mitochondrial, or Archea DNA targeted). PCR reactions were performed in 25 μL mixture containing 5 μL of 5 × GC Buffer, 0.75 μL of KAPA dNTP Mix, 0.5 μL of KAPA HiFi HotStart DNA Polymerase, 1.5 μL of each primer (10 pM) and 5 μL of purified product. PCR reaction cycling included 95 °C for 3 min, followed by 8 cycles at 95 °C for 30 s, 55 °C for 30 s, and 72 °C for 30 s and a final extension at 72 °C for 5 min. The amplicons were subsequently purified by AMPure XP beads to clean up the final library before quantification. Last, purified amplicons were pooled in equimolar and paired-end sequenced (2 × 250) on an Illumina MiSeq platform according to the standard protocols. The sequencing resulted in 16S rRNA bacterial fragments confirming the specificity of our primers as predicted in silico and excluding any artifact due to the unwanted amplification of eukaryotic DNA. A standard curve for quantifying 16S rRNA gene copies was established using a ten-fold serial dilution, ranging from 1 to 107 copies per reaction, of plasmid DNA containing the complete 16S rRNA gene from Escherichia coli BL21 strain. Amplifications of samples and standard dilutions were performed in triplicates on a real-time PCR system (LifeTechnologies, Carlsbad, CA, USA). The quality of the amplified product was assessed by melting curves. One donor was excluded from the qPCR analysis for technical reasons.

### 2.5. Statistical Analysis

Fast Length Adjustment of SHort reads (FLASH) was used to merge paired-end reads from next-generation sequencing [34]. Low quality reads were filtered by fastq_quality_filter (-p 90 -q 25 -Q33) in FASTX Toolkit 0.0.14 and chimera reads were removed by USEARCH 64 bit v8.0.1517. The number of reads for each sample was normalized based on the smallest size of samples by random subtraction. OTUs were aligned by UCLUST algorithm with a 97% identity and taxonomically classified using the SILVA 16S rRNA database v128. Alpha and beta diversities were generated in the Quantitative Insights Into Microbial Ecology (QIIME) and calculated based on weighted and unweighted Unifrac distance matrices [35]. The output matrix containing the relative abundance of OTUs per sample was analyzed using the Linear Discriminant Analysis Effect Size (LEfSe) algorithm with an alpha threshold of 0.05 and an effect size threshold of 2.0. Statistical evaluations, including nonparametric Mann–Whitney and Kruskal–Wallis tests, followed by Dunn’s multiple comparison tests and Spearman’s correlations, were performed utilizing PRISM software (Version 6.05, GraphPad, Inc., La Jolla, CA, USA) and R environment (Version 3.1.2, https://cran.r-project.org/ bin/windows/base/old/3.1.2/, accessed on 9 May 2024.). Data are presented as means ± standard deviations (SD) are illustrated in the figures (* *p*< 0.05, ** *p*< 0.01, *** *p* < 0.001, **** *p* < 0.0001). Following rigorous quality control (filtering of low-quality sequences and removal of chimeras), the median (range) number of raw high-quality NGS reads per sample was 203,602 (48,129–321,419) sequences. To ensure comparability across all downstream analyses, sequencing depths for all samples were uniformly standardized to 48,129 sequences per sample via random subsampling (i.e., the minimum number of sequences found across all samples). This uniform depth was used for all subsequent analyses.

## 3. Results

### 3.1. General Characteristics of Study Participants

A cohort of 9 patients with severe coronary artery stenosis and 18 patients with mild to moderate coronary artery stenosis were included in the study. The demographic breakdown, as detailed in Table 1, included 15 males and 12 females, aged 41–84 years (averaged at 61.56 ± 11.42 years). Patients with severe stenosis exhibited significantly higher admission Troponin T levels and increased high-sensitivity C-reactive protein (hs-CRP) compared to those with mild to moderate stenosis (*p* < 0.05). Of the 27 patients, 11 (40.7%) had diabetes mellitus, including 6 (33.3%) in the mild-to-moderate stenosis group and 5 (55.6%) in the severe stenosis group (*p* = 0.411; Table 1). No significant differences were observed between the two groups in terms of gender, age, body mass index, smoking, hypertension, diabetes, renal function and blood lipid indexes (Table 1).

### 3.2. Blood from CHD Patients Contains Bacterial DNA That Is Differentially Distributed Among Blood Fractions

**Figure 1:**
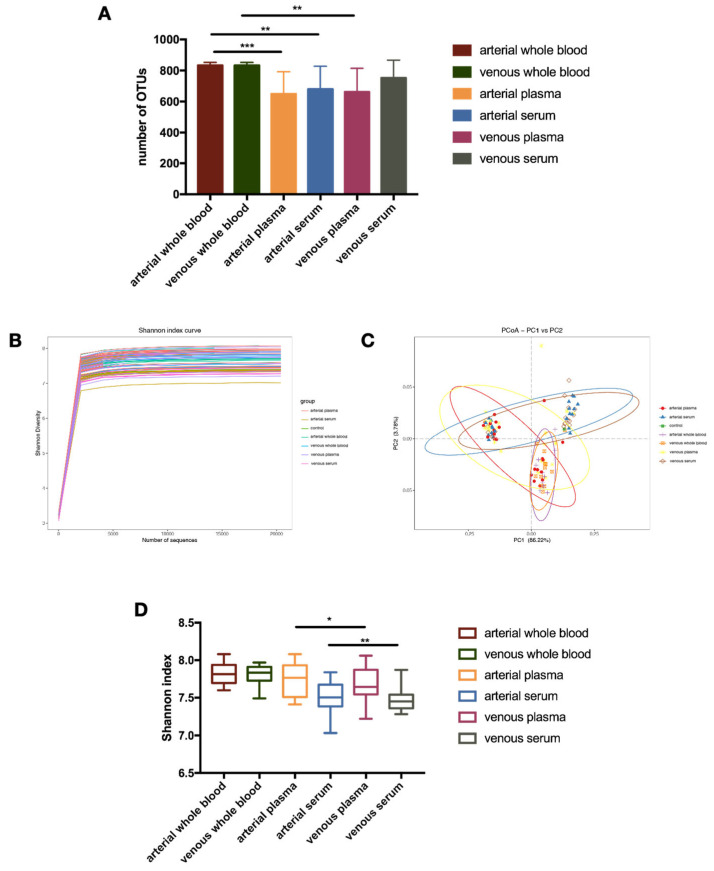
Comparative Analysis of Bacterial DNA Distribution and Diversity in Blood Fractions. (**A**) Number of OTUs identified in arterial and venous blood fractions. (**B**) Shannon diversity index across different blood fractions. (**C**) PCoA of bacterial DNA profiles using Generalized UniFrac distances. (**D**) Boxplot of Shannon index for arterial and venous whole blood, plasma, and serum. Notes: * indicates a *p*-value < 0.05, ** indicates a *p*-value < 0.01, and *** indicates a *p*-value < 0.001.

Analysis of 16S rRNA gene copies across various fractions revealed the presence of bacterial DNA in whole blood, serum, and plasma from both arteries and veins. The operational taxonomic units (OTUs) count was notably higher in whole blood compared to serum and plasma in arterial and venous samples (Figure 1D). Although we observed a tendency for higher OTU counts in serum compared to plasma, this difference was not statistically significant (Figure 1A). As shown in Figure 1A, the bacterial DNA content was notably lower in serum samples compared to arterial and venous blood fractions.

### 3.3. Patients’ Arterial and Venous Serum Have Lower Blood Bacterial 16s rRNA Diversity

We conducted high-throughput 16S targeted metagenomic sequencing to analyze the taxonomic diversity and profile of bacterial DNA in blood. Following clustering in OTUs and taxonomic assignment, the Shannon diversity index (illustrated at the OTU level in Figure 1B) revealed a significantly higher bacterial diversity in the plasma fractions of both arteries (*p* = 0.05) and veins (*p* = 0.01) compared to serum (Figure 1B,D). Additionally, Principal Coordinate Analysis (PCoA) utilizing Generalized UniFrac distance metrics (alpha value of 0.32, Figure 1C) revealed statistically significant differences among plasma and serum study groups (Figure 1D).

### 3.4. Similarities and Differences in Bacterial Profiles Between Fractions as Assessed by 16S Targeted Metagenomic Sequencing

**Figure 2:**
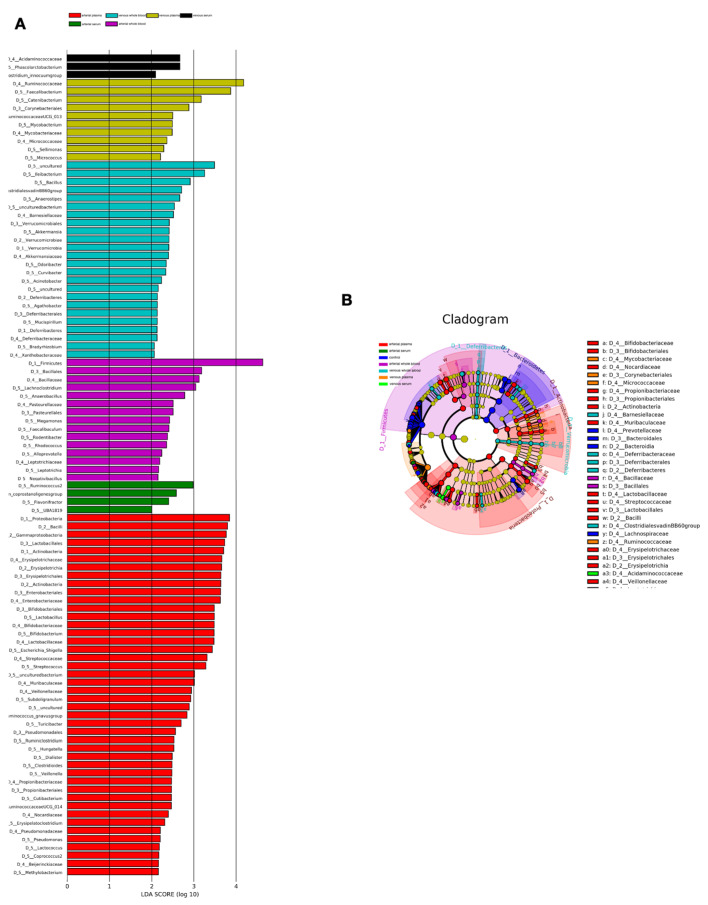
Differential Bacterial Enrichment in Blood Fractions. (**A**) Histogram of LDA scores indicating the most differentially abundant taxa between blood fractions with a score threshold of ≥2. (**B**) Cladogram visualizing the phylogenetic distribution of significantly enriched bacterial taxa across blood fractions based on LEfSe analysis.

The specific bacterial enrichment across blood fractions is illustrated in histograms (Figure 2A) and cladograms (Figure 2B), derived from an LDA score ≥ 2 in pairwise comparisons. The LEfSe analysis revealed a higher abundance of *phylum Firmicutes* (*Bacillota*), the families *Bacillaceae* and *Pasteurellaceae*, and the genera *Lachnoclostridium* and *Anaerobacillus* in arterial blood than in venous blood. Higher proportions of the genera *Ileibacterium*, *Bacillus, ClostridialesvadinBB60*, *Anaerostipes*, *Barnesiellaceae*, *Verrucomicrobiales*, *Akkermansia and Deferribacteraceae* were observed in arterial blood. At the same time, there are striking differences between fractions: the *Acidaminococcaceaethe*, *Phascolarctobacterium* and *Clostridium innocuum Sphingobacteria* were mostly found in venous serum, and *Ruminococcaceae*, *Mycobacteriaceae*, *Faecalibacterium*, *Catenibacterium*, *Mycobacterium* mostly in venous plasma. In patient, arterial serum circulating microbiota exhibited an enrichment of *Coprostanoligenes*, *Ruminococcus* and *Flavonifractor* at the genus level, *Proteobacteria* and *Acfinobacteria* at the phylum level, *Bacilli*, *Gammaproteobacteria*, and *Erysipelotrichia at the class level*, *Lactobacillales*, *Enterobacteriales*, and *Bifidobacteriales* at the order level, *Erysipelotrichaceae*, *Streptococcaceae*, *Lactobacillaceae*, *Bifidobacteriaceae* and *Muribaculaceae* at the family level, *Veillonella*, *Erysipelatoclostridium and Bifidobacterium* at the genus level were specifically enriched in arterial plasma.

Additionally, Table 2 presents the average relative abundance of Firmicutes and Bacteroidetes across the six blood sample groups (arterial whole blood, venous whole blood, arterial plasma, venous plasma, arterial serum, venous serum). Table 3 incorporates statistical tests for differences in the absolute abundance of Firmicutes and Bacteroidetes across groups using the Metastats algorithm, including pairwise comparison tests and *p*-values for each taxonomic unit within these phyla.

## 4. Discussion

Prior research indicates that various diseases, including inflammatory conditions, sepsis, cirrhosis, cardiovascular disorders, and diabetes, facilitate bacterial translocation into tissues originating from the gut [5,7,8,9,10,11,12,13,14,15,16,36]. Meanwhile, many groups have reported the successful culture and microscopic observation of numerous bacteria from the blood of healthy individuals. In addition, numerous studies have cultured and observed bacteria in the blood of healthy individuals, as well as patients with tumors, cardiovascular disease, liver disease, or Parkinson’s disease [37,38]. Due to advances in high-throughput sequencing technology and optimization of specific processes for specific metagenomics, we have succeeded in quantifying and characterizing the presence of bacterial DNA in the whole blood, serum and plasma in arteries and veins of patients with CHD, which was consistent with previous studies. However, there have been few exhaustive analyses of the microbiome present in arterial and venous blood until now. Our findings reveal a significantly higher number of OTUs in whole blood compared to serum and plasma across both arterial and venous samples. However, no significant difference was observed in OTU numbers between arterial and venous blood or between plasma and serum.

Microorganisms cultured from critically ill patients have shown higher positive rates in arterial than in venous blood samples [39]. High-throughput sequencing studies in animal and human models further suggest molecular differences between arterial and venous blood, including distinct patterns of circulating miRNAs [40,41]. These data indicate that arterial and venous blood are not compositionally identical. In our cohort, Shannon diversity did not differ significantly between arterial and venous blood, but we observed differential enrichment of specific taxa: genera such as *Ileibacterium*, *Bacillus*, *Clostridiales vadinBB60*, *Anaerostipes*, *Barnesiellaceae*, *Verrucomicrobiales*, *Akkermansia* and *Deferribacteraceae* were more abundant in venous blood, whereas *Firmicutes*, *Bacillaceae*, *Pasteurellaceae*, *Lachnoclostridium* and *Anaerobacillus* were more enriched in arterial blood. Taken together, Shannon diversity did not differ significantly, we interpret these arterial–venous patterns only as hypothesis-generating trends of selective enrichment rather than definitive differences in overall diversity. These patterns may be influenced by DNA clearance, coagulation and transient bacteremia, medication and other confounders, as well as low-biomass sequencing limitations, and thus should be interpreted cautiously and confirmed in larger studies with stricter controls.

Previous study has demonstrated that a diversified microbiome exists in healthy blood [16]. Blood from healthy donors contains bacterial DNA that is differentially distributed between blood fractions and individuals. The buffy coat harbors most blood bacterial DNA, while red blood cells (RBCs) exhibit higher bacterial DNA levels than plasma. However, no studies have confirmed the distinction in microbial characteristics between plasma and serum. Our investigation contributes to this gap by showing that serum tended to have a higher OTU count than plasma in both arterial and venous samples, although this difference was not statistically significant. In contrast, plasma demonstrated greater bacterial diversity, as reflected by higher Shannon diversity indices in both arteries and veins. This pattern was supported by Principal Coordinate Analysis (PCoA) based on Generalized UniFrac distances, which revealed statistically significant differences in microbial community structure between plasma and serum groups. Taken together, these findings suggest that compositional shifts between plasma and serum are more pronounced at the level of α- and β-diversity than in simple OTU counts. Previous work has shown that plasma and serum differ in their biochemical composition, including lipids, amino acids, and other metabolites, and that preanalytical factors can influence these profiles [42,43,44,45,46]. These studies support the notion that plasma and serum are not compositionally identical and may partly underlie the distinct bacterial DNA diversity and community structure we observed. However, our study was not designed to dissect these mechanisms, and the potential contributions of sample processing and coagulation remain speculative.

We also analyzed similarities and differences in bacterial profiles across various blood components through 16S targeted metagenomic sequencing. Significant abundance differences were observed among various blood components, with 15 genera in arterial whole blood, 22 in venous whole blood, 43 in arterial plasma, 10 in venous plasma, 4 in arterial serum, and 3 in venous serum. Many of the detected bacterial DNA signals corresponded to taxa typically associated with intestinal, oral, or skin microbiota, such as *Firmicutes*, *Bacillales*, *Phascolarctobacterium*, *Lactobacillus*, *Bacteroides*, and *Streptococcus*, suggesting a potential link between gut- or barrier-associated communities and circulating bacterial DNA in CHD patients. Our results are consistent with the findings of pioneers like Amar et al. [47]. Zhou et al. [12] reported for the first time the higher microbial richness and diversity in the systemic microbiome of STEMI patients. More than 12% of post-STEMI blood bacteria were dominated by intestinal microbiota (*Lactobacillus*, *Bacteroides*, and *Streptococcus*). Previous studies have proposed that translocation of gut-derived bacteria into the systemic circulation may be related to intestinal mucosal injury and gut barrier failure, particularly in acute coronary syndromes, but these mechanisms were not directly assessed in our study. Our findings are broadly in line with these reports at the phylum and genus level; however, unlike these studies, we did not assess clinical outcomes or perform functional analyses, and our small sample size and lack of healthy controls limit direct comparison of effect sizes or disease-specific signatures. The 16S rRNA gene sequencing approach used in our study cannot distinguish viable bacteria from dead bacteria or cell-free bacterial DNA (cfDNA), so the signals detected here should be interpreted as a blood bacterial DNA profile rather than definitive evidence of a viable blood bacterial DNA profile. In CHD patients, intestinal mucosal injury and gut barrier dysfunction have been hypothesized to be driven by ischemic stress (e.g., compromised LV function and intestinal hypoperfusion), but these processes were not evaluated in our cohort. Meanwhile, some bacteria may come from the oral cavity or skin, such as *Anaerobacillus* and *Cutibacterium*. Dietary factors, including artificial sweeteners, have been shown to alter gut microbial composition and intestinal permeability, potentially exacerbating microbial translocation and systemic inflammation, which may represent an underappreciated modifier of circulating microbiota profiles in cardiovascular disease [48]. The higher bacteria richness and distinct microbial community drove us to explore the origin of bacteria.

In our cohort, more than one-third of patients had comorbid diabetes mellitus, a well-recognized risk factor for CHD and a potential modifier of the circulating microbiome. Although the proportion of diabetes was higher in the severe stenosis group, this difference did not reach statistical significance, probably due to the limited sample size. Larger studies are needed to clarify the impact of diabetes on blood microbiota profiles in this setting. We acknowledge that the observed differences in microbiota composition may be influenced by factors such as DNA clearance, contamination, coagulation artifacts, transient bacteremia, medication, diet, sequencing artifacts, access site contamination, and normal physiological variations, all of which should be considered when interpreting the results.

Overall, this exploratory study provides a first systematic comparison of bacterial DNA profiles across arterial and venous whole blood, plasma, and serum in patients with CHD. The differences observed between arterial and venous compartments and between plasma and serum may reflect variation in the generation, circulation, or clearance of bacterial DNA, but these possibilities remain speculative and were not directly tested in our study. These findings lay the groundwork for future research into how microbiota interact with host physiology and contribute to disease mechanisms. Further studies integrating approaches such as metabolomics or immunological analyses are needed to explore these interactions in more detail, potentially identifying new biomarkers or therapeutic targets for CHD.

Several limitations should be noted. Firstly, the 16S rRNA gene sequencing approach cannot distinguish viable bacteria from dead bacteria or cell-free bacterial DNA. Therefore, we cannot determine whether the detected bacterial DNA originated from live microorganisms. Secondly, we did not include reagent-only negative extraction controls or sequencing blanks. The absence of these controls limits our ability to distinguish true biological signals from background contamination, which could be influenced by kitome DNA or environmental contaminants. Thirdly, the small sample size limits the statistical power, and we did not perform formal power calculations, as this study was exploratory. Future studies should use larger sample sizes and include power calculations to improve statistical robustness. Fourthly, we did not directly compare microbial composition between the mild and severe stenosis groups. we did not include a healthy control group, which limits the ability to define disease-specific microbiome signatures. Future research should include healthy controls to validate these findings and explore the impact of stenosis severity on microbiota composition. Fifthly, we did not conduct functional studies to investigate how microbiota differences contribute to disease mechanisms. Future studies should integrate functional analyses to better understand the role of microbiota in coronary heart disease. Additionally, the sequencing depth (48,129 reads/sample) limits the detection of low-abundance species, which may affect alpha diversity estimation. However, observed differences in bacterial species (gamma-diversity) could reflect genuine biological variation among blood compartments. In low-biomass studies, limited sequencing depth can lead to over- or underestimation of diversity, and biological interpretation should consider effect sizes [19]. Therefore, our findings should be considered preliminary. Since this study focused on relative microbiome composition, we did not perform qPCR to calculate absolute 16S rRNA gene copy numbers. Future studies should incorporate absolute quantification for a more comprehensive assessment of bacterial burden.

## 5. Conclusions

Taken together, our study provides preliminary evidence for differences in bacterial DNA profiles between arterial and venous blood and between plasma and serum in patients with coronary heart disease. These findings should be regarded as hypothesis-generating and interpreted with caution in light of the small sample size and methodological limitations, including the challenges of low-biomass microbiome analysis and contamination control. Future studies with larger cohorts, rigorous negative controls, viability assays, and functional readouts will be essential to determine whether these patterns reflect biologically meaningful phenomena or technical or physiological artifacts.

## Figures and Tables

**Table 1:** Characteristics of patients with mild to moderate/severe coronary artery stenosis.

	Coronary Artery Stenosis	Coronary Artery Stenosis	*p* Value
	Mild to Moderate (*n* = 18)	Severe (*n* = 9)	
Gender (female)	10 (55.56%)	2 (22.22%)	0.217
Age (year)	61.83 ± 8.83	61 ± 16.05	0.887
Body mass index (kg/m^2^)	25.08 ± 3.12	26.42 ± 4.65	0.38
Current smoking	4 (22.22%)	3 (33.33%)	0.635
Hypertension	10 (55.56%)	8 (88.89%)	0.193
Diabetes	6 (33.33%)	5 (55.56)	0.411
Systolic blood pressure	134 ± 14.64	135.56 ± 10.78	0.307
Diastolic blood pressure	83.56 ± 12.38	74.78 ± 15.56	0.123
Blood biochemical tests			
Troponin T_admission (ng/mL)	0.01 ± 0.01	0.09 ± 0.12	0.016 *
urea (mmol/L)	4.65 ± 1.29	8.39 ± 4.19	0.168
uric acid (umol/L)	368.28 ± 78.4	376 ± 76.54	0.81
creatinine (umol/L)	68.04 ± 14.06	194.54 ± 34.99	0.31
eGFR (ml/min/1.73 m^2^)	91.31 ± 10.64	77.24 ± 34.83	0.267
Glucose (mmol/L)	6.52 ± 2.75	8.35 ± 4.19	0.185
Total cholesterol (mmol/L)	4.17 ± 1.03	3.92 ± 1.82	0.646
Triglyceride (mmol/L)	1.84 ± 1.32	1.38 ± 0.85	0.35
High-density lipoprotein (mmol/L)	1.23 ± 0.51	1.26 ± 0.56	0.867
Low-density lipoprotein (mmol/L)	2.32 ± 0.90	2.29 ± 1.18	0.941
hs-CRP (mg/L)	2.67 ± 4.83	16.99 ± 27.67	0.039 *

Abbreviations: eGDR, estimated glomerular filtration rate; hs-CRP, high-sensitivity C-reactive protein; * *p* < 0.05.

**Table 2:** Average Relative Abundance of Firmicutes and Bacteroidetes.

	Firmicutes			Bacteroidetes		
	Mean	Variance	Stderr	Mean	Variance	Stderr
arterial plasma	0.6684	0.0018	0.0129	0.2152	0.0005	0.0069
arterial serum	0.6203	0.0002	0.0040	0.3060	0.0002	0.0037
venous plasma	0.6580	0.0010	0.0093	0.2416	0.0009	0.0085
venous serum	0.6230	0.0005	0.0067	0.2970	0.0003	0.0051
arterial whole blood	0.5870	0.0029	0.0156	0.1827	0.0014	0.0108
venous whole blood	0.5894	0.0034	0.0168	0.1932	0.0018	0.0123

**Table 3:** Pairwise Comparison of Sequence Counts for Firmicutes and Bacteroidetes Across Sample Groups.

		Arterial Plasma	Arterial Serum	Venous Plasma	Venous Serum	Arterial Whole Blood	Venous Whole Blood
Firmicutes	arterial plasma	/	0.0040	0.5205	0.0030	0.0020	0.0040
	arterial serum	0.0040	/	0.0010	0.7073	0.0519	0.0909
	venous plasma	0.5205	0.0010	/	0.0060	0.0010	0.0020
	venous serum	0.0030	0.7073	0.0060	/	0.0380	0.0759
	arterial whole blood	0.0020	0.0519	0.0010	0.0380	/	0.9111
	venous whole blood	0.0040	0.0909	0.0020	0.0759	0.9111	/
Bacteroidetes	arterial plasma	/	0.0010	0.0260	0.0010	0.0170	0.1329
	arterial serum	0.0010	/	0.0010	0.1708	0.0010	0.0010
	venous plasma	0.0260	0.0010	/	0.0010	0.0040	0.0040
	venous serum	0.0010	0.1708	0.0010	/	0.0010	0.0010
	arterial whole blood	0.0170	0.0010	0.0040	0.0010	/	0.5425
	venous whole blood	0.1329	0.0010	0.0040	0.0010	0.5425	/

## Data Availability

The data that support the findings of this study are not publicly available due to patient privacy concerns and institutional restrictions. They may be available from the corresponding author upon reasonable request and subject to institutional approval.

## Abbreviations

The following abbreviations are used in this manuscript:

BAs	bile acids
CHD	Coronary heart disease
eGDR	estimated glomerular filtration rate
FLASH	Fast Length Adjustment of SHort reads
hs-CRP	high-sensitivity C-reactive protein
LPC	lyso-phosphatidylcholines
LPE	lyso-phosphatidylethanolamines
MI	myocardial infarction
OTUs	operational taxonomic units
PCoA	Principal Coordinate Analysis
QIIME	Quantitative Insights Into Microbial Ecology
SCFAs	short-chain fatty acids
TMAO	trimethylamine N-oxide
